# Long-Term Patient-Reported Outcomes After Radiofrequency Ablation and Cryoballoon Ablation for Paroxysmal Atrial Fibrillation: The Effect of Additional Ablations

**DOI:** 10.3390/jcdd11120385

**Published:** 2024-11-30

**Authors:** Ibrahim Antoun, Ahmed I. Kotb, Zakkariya Vali, Ahmed Abdelrazik, Ivelin Koev, Kassem Safwan, Edward Y. M. Lau, Riyaz Somani, Ghulam André Ng

**Affiliations:** 1 Department of Cardiovascular Sciences, Clinical Science Wing, University of Leicester, Glenfield Hospital, Groby Road, Leicester LE3 9QP, UK; z.vali@leicester.ac.uk (Z.V.); el203@leicester.ac.uk (E.Y.M.L.); riyaz.somani@uhl-tr.nhs.uk (R.S.); 2 Department of Cardiology, University Hospitals of Leicester NHS Trust, Glenfield Hospital, Leicester LE3 9QP, UK; aiamk1@leicester.ac.uk (A.I.K.); ahmed.abdelrazik@leicester.ac.uk (A.A.); koev.vn@gmail.com (I.K.); kassem.safwan@uhl-tr.nhs.uk (K.S.); 3 National Institute for Health Research Leicester Research Biomedical Centre, Leicester LE5 4PW, UK

**Keywords:** atrial fibrillation, catheter ablation, quality of life, radiofrequency ablation, cryoballoon ablation

## Abstract

Background: pulmonary vein isolation (PVI) for paroxysmal atrial fibrillation (PAF) improves health-related quality of life (QoL). This study compares QoL improvement after radiofrequency ablation (RF) and cryoballoon ablation (cryo) and assesses additional ablations’ role in QoL improvement. Methods: we evaluated the QoL of consecutive patients with first-time RF and cryo for PAF between January 2017 and June 2019. A combined EQ-VAS, AFEQT, and EQ-5D-3L paper questionnaire was sent to patients at baseline, 12, and 30 months after the procedure. Procedure and patient details were collected from medical notes. Results: the analysis included 207 patients, of which 127 (61%) had RF and 144 (70%) were males. RF patients had more additional ablations (52 [41%] versus 22 [28%], *p* = 0.01). There was a significant improvement from baseline to 12 months post-RF in AFEQT (43 ± 9 to 83 ± 7.8, *p* < 0.001), EQ-5D-3L (−0.01 ± 0.01 to 1.1 ± 0.02, *p* < 0.001), and EQ-VAS (51 ± 8 to 77 ± 13, *p* = 0.01). Similarly, an improvement at 12 months was observed after cryo in AFEQT (55 ± 11 to 77 ± 9, *p* < 0.001), EQ-5D-3L (−0.04 ± 0.03 to 1.3 ± 0.03, *p* < 0.001), and EQ-VAS (56 ± 7 to 85 ± 9, *p* = 0.01). QoL improvement was similar between RF and cryo. Additional ablations provided no additional QoL improvement compared to patients with PVI alone. Conclusions: Patients undergoing first-time PVI for PAF, RF, and cryo showed similar QoL improvement at 12 months, which was sustained at 30 months. Additional ablations did not provide further QoL benefits.

## 1. Introduction

Health-related quality of life (QoL) is a subjective measure of a patient’s health perceptions and functional status [1]. Atrial fibrillation (AF) is a chronic progressive disease and the most common sustained arrhythmia seen in clinical practice. Although rarely life-threatening, AF is associated with increased morbidity and mortality, predominantly through a higher risk of left ventricular dysfunction, thromboembolic events, and significant impairments in QoL and functional capacity [2,3,4]. AF, with its unpredictable episodes of abnormal heart rhythm, affects everyday life by causing physical, mental, and social limitations. Patients also experience worry and anxiety about financial concerns due to the loss of workdays and the cost of managing AF. There has been a recent interest in assessing QoL in AF patients worldwide [5].

Pulmonary vein isolation (PVI) for AF provides an effective way to maintain sinus rhythm when antiarrhythmic drugs (AADs) are ineffective, contraindicated, or not tolerated (5, 6). Modern AF ablation procedures typically use advanced-generation AF ablation technologies, such as dedicated ablation catheters capable of achieving PVI with a single ablation lesion or advanced-generation focal radiofrequency (RF) catheters capable of real-time quantitative assessments of catheter–tissue contact force. Point-by-point radiofrequency ablation (RF) and cryoablation (cryo) are the two main methods to isolate pulmonary veins (PVs). Cryo uses a one-shot technique utilising cryo energy, while RF uses a point-by-point approach to isolate PVs using radiofrequency energy. Two landmark studies suggested QoL improvements following PVI [6,7]. Also, five studies proposed that PVI is superior to drug therapy in QoL improvement (14–18). However, this superiority decreased over time, probably due to AF recurrence in PVI patients (19). When compared directly, CIRCA-DOSE and Fire and Ice studies demonstrated QoL improvement in PAF patients after RF and cryo [8,9]. However, the effect of additional ablations outside of PVs was not explored. This study compares long-term QoL improvement between RF and cryo with and without additional ablations.

## 2. Material and Methods

### 2.1. Patient Selection and Data Collection

This retrospective observational cohort study included consecutive patients who completed their first PVI for PAF between January 2017 and June 2019 in Glenfield Hospital, a tertiary cardiology centre in Leicester, UK. Patients who had received previous ablation procedures, patients with persistent AF, and patients who did not complete their 12- and 30-month follow-ups were also excluded. PVI was conducted by contact force RF, or second-generation catheters were used for cryo. All involved patients had complete PVI with confirmed bidirectional block. Recurrence was defined as AF seen on the 12-leads electrocardiogram (ECG) or AF lasting 30 s or more on Holter monitoring.

Patient demographics and medication details were obtained electronically by examining clinic appointment letters, which provided clinical information, medications, ablation details, and follow-up appointments. The study was conducted as part of an audit approved by the University Hospitals of Leicester NHS Trust (reference number: 35479-ia196). It was reported according to the STROBE guidelines [10].

### 2.2. Ablation Details

All procedures were performed as first-time ablations in a single setting. In the RF procedure, a circular mapping catheter was deployed in the superior and inferior pulmonary veins (PVs). Circumferential ablation of left-sided and right-sided ipsilateral PVs was conducted, guided by three-dimensional left atrial mapping (CARTO3, Biosense Webster, Irvine, CA, USA). A 3.5 mm irrigated-tip ablation catheter (ThermoCool SmartTouch Catheter, Biosense Webster, Diamond Bar, CA, USA) was used with ablation index guidance. Post-procedure, dormant conduction in the PVs was assessed using rapid adenosine triphosphate injection. In the cryo procedures, a long sheath (8.5 Fr SL0, Abbott Laboratories, Chicago, IL, USA) was transitioned to a steerable sheath (FlexCath, Medtronic, Minneapolis, MN, USA), allowing for the insertion of a second-generation cryoballoon (28 mm) into the left atrium over an inner-lumen circumferential mapping catheter (Achieve, Medtronic). The cryoballoon was frozen at the superior and inferior left and right PVs’ ostium to achieve a bidirectional conduction block as the endpoint of the PVI procedure.

### 2.3. QoL Questionnaires

The paper form combined three questionnaires: the AFEQT (the AF effect on quality of life survey), EQ-5D, and EQ-VAS. QoL paper questionnaires were administered at baseline, 12, and 30 months after ablation, similar to the Fire and Ice study [8]. The questionnaires are shown in the Supplementary Material.

### 2.4. AFEQT

The AFEQT (atrial fibrillation effect on quality of life) questionnaire contained 20 items and included six domains: treatment satisfaction and concerns, emotional, physical, and social functioning. Each domain is answered using a 0–7 scale, with 0 demonstrating severe disability and 7 demonstrating no disability. It was explicitly designed for AF patients and assesses ablation satisfaction. It was also proven reliable with a reliability coefficient > 0.8 [11] and was strongly linked with symptom severity [12].

The AFQT questionnaire was scored out of 100%, with 100% representing the best QoL possible and 0% representing the worst QoL possible.

### 2.5. EQ-5D-3L

This questionnaire was developed in 1987 by the EuroQoL group. It aimed to devise generic health measurements used in clinical investigations [13]. It consisted of five questions about depression/anxiety, discomfort/pain, daily activities, self-care, and mobility [14]. Each question had three options, depending on degree. It was proven valid and reliable in multiple populations [15,16] and cardiovascular conditions [17]. The levels can be combined to calculate a UK-specific summary index, as developed by Dolan in 1997 [18].

### 2.6. EQ-VAS

The EQ-VAS is usually displayed as a vertical line, with clearly labelled endpoints at 0 and 100. The respondent indicates a point on the line representing their current health status, with 100 representing the best possible health state and 0 representing the worst possible health state.

### 2.7. Outcomes

The outcomes of this study include QoL:

1. Comparison of QoL improvement in RF and cryo at 12 and 30 months.
2. QoL improvement in RF and cryo without additional ablations and the effect of additional ablations on QoL improvement.
3. The effect of successful ablation on QoL improvement.
4. Predictors of QoL improvement.

### 2.8. Statistical Analysis

Categorical variables were expressed as frequency and percentage. The mean ± standard error of the mean was adopted to describe continuous parametric data. Pearson’s χ2 or Fisher’s exact test was used for categorical variables between groups. Student’s *t*-tests and Mann–Whitney-U tests were used to compare continuous variables, including P wave parameters, between the groups depending on the normality of the distribution. A multiple linear regression model was conducted with an outcome of a change in the EQ-VAS with the interaction term between ablation modality, additional ablations, procedure outcome and QoL improvement. A linear mixed-effects model was used to analyse the repeated measures of QoL over time (baseline, 12 months, and 30 months). This model allowed for random intercepts to account for individual variability and included demographics and ablation characteristics.

A two-sided *p*-value < 0.05 was considered statistically significant. Statistical analyses were performed using GraphPad Prism V9.3 (San Diego, CA, USA).

## 3. Results

### 3.1. Demographics and Ablation Details

Patient characteristics and ablation details are shown in Table 1. The final analysis included 207 patients, of which 127 (65%) had RF and 144 (70%) were males. The mean age was 62 ± 4.2 years. When comparing RF and cryo, demographics were similar. RF patients had more additional ablations (41% vs. 28%, *p* = 0.01). All additional ablations in cryo were at the roofline using a cryo catheter. Of the additional RF ablations, posterior wall ablation occurred in six (12%) and complex fractionated atrial electrogram (CFAE) in four (8%) patients. Coronary sinus and mitral valve isthmus ablation occurred in 6 (12%) patients, and 36 (69% of additional ablations) had roofline ablations. The overall success rate was 74% and 68% at 12 and 30 months, respectively. There was no difference in success rate between RF and cryo, nor the rate and rhythm control drugs used. Also, the success rates at 12 and 30 months were not different for patients (77% vs. 74%, *p* = 0.63 and 69% vs. 70%, *p* = 0.93, respectively).

### 3.2. QoL Scores

**Figure 1:**
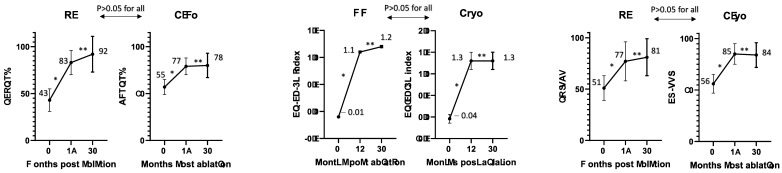
Comparison of quality of life scores between radiofrequency ablation and cryoballoon ablation at follow-ups. Cryo: cryoballoon. * Represents *p* < 0.05. ** Represents *p* > 0.05.

EQ-5D-3L, AFEQT, and EQ-VAS scores at baseline and follow-ups in RF and cryo were compared in Figure 1 There was a significant improvement from baseline to 12 months post-RF in AFEQT (43 ± 9 to 83 ± 7.8, *p* < 0.001), EQ-5D-3L (−0.01 ± 0.01 to 1.1 ± 0.02, *p* < 0.001), and EQ-VAS (51 ± 8 to 77 ± 13, *p* = 0.01). Similarly, the improvement at 12 months was observed after cryo in AFEQT (55 ± 11 to 77 ± 9, *p* < 0.001), EQ-5D-3L (−0.04 ± 0.03 to 1.3 ± 0.03, *p* < 0.001), and EQ-VAS (56 ± 7 to 85 ± 9, *p* = 0.01). However, there was no significant QoL improvement between 12 and 30 months after both ablation modalities across all questionnaires. The QoL improvement was similar between RF and cryo in all questionnaires at 12 months (AFEQT: *p* = 0.72, EQ-5D: *p* = 0.31, EQ-VAS: *p* = 0.62) and 30 months (AFEQT: *p* = 0.72, EQ-5D: *p* = 0.27, EQ-VAS: *p* = 0.11).

### 3.3. QoL Improvement Without Additional Ablations

**Figure 2:**
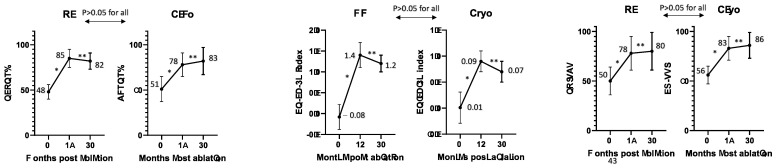
Comparing the effect of additional ablation outside the pulmonary veins on the quality of life in radiofrequency and cryoballoon ablation. Cryo: cryoballoon. * Represents *p* < 0.05. ** Represents *p* > 0.05.

After excluding 52 RF and 22 cryo patients who had additional ablations, patients who had successful ablation were similar between RF and cryo (61 [81%] versus 44 [76%], *p* = 0.15). QoL results are demonstrated in Figure 2 Similarly to the total cohort, patients who had PVI only had improvements in QoL scores across all questionnaires in RF from baseline to 12 months post-RF in AFEQT (48 ± 7 to 85 ± 6, *p* < 0.001), EQ-5D-3L (−0.16 ± to 1 ± 0.01, *p* < 0.001), and EQ-VAS (49 ± 4.6 to 79 ± 2.1, *p* < 0.001). Similarly, the improvement at 12 months was observed after cryo in AFEQT (55 ± 4.7 to 78 ± 2.2, *p* = 0.03), EQ-5D-3L (−0.08 ± 0.02 to 1.4 ± 0.02, *p* < 0.001), and EQ-VAS (50 ± 7 to 78 ± 8, *p* < 0.001). However, there was no significant QoL improvement between 12 and 30 months after both ablation modalities in all questionnaires. RF and cryo did not demonstrate differences in QoL scores across all questionnaires at 12 months (AFEQT: *p* = 0.12, EQ-5D: *p* = 0.72, EQ-VAS: *p* = 0.36), and 30 months (AFEQT: *p* = 0.61, EQ-5D: *p* = 0.31, EQ-VAS: *p* = 0.6).

### 3.4. The Effect of Successful Ablation on QoL Maintenance

Multiple linear regression investigated the relationship between QoL improvement and several predictors, including the ablation method, additional ablations, and SR maintenance at 12 months.

The overall regression model was not statistically significant (F(3, 146) = 1.52, *p* = 0.21), indicating that the predictors did not explain a significant proportion of the variance in job satisfaction. The adjusted R^2^ was 0.05, suggesting that the model accounted for only 5% of the variability in QoL improvement. Individually, none of the predictors were found to predict QoL improvement. RF vs. cryo (B = 0.05, standard error [SE] = 0.03, *p* = 0.12), additional ablations (B = −0.01, SE = 0.04, *p* = 0.76), and maintenance of SR at 12 months (B = 0.02, SE = 0.05, *p* = 0.65) did not have statistically significant relationships with QoL improvement. Multicollinearity assumptions were checked using variance inflation factors, and they were all below two, indicating that multicollinearity was not a concern. Additionally, residual diagnostics showed no significant deviations from normality, linearity, or homoscedasticity.

### 3.5. QoL Improvement Predictors

The linear mixed-effects model revealed a significant effect of time on QoL, indicating that patients’ QoL improved over the study period, regardless of the ablation method used. However, the ablation method (cryo vs. RF) was not a significant predictor of QoL, and there was no significant interaction between time and the ablation method.

The effect of time was significant: F(2, 150) = 6.03, *p* = 0.01. Post hoc comparisons showed that QoL improved significantly from the baseline to 12 months (*p* < 0.01) and from baseline to 30 months (*p* < 0.01), with no significant difference between 12 months and 30 months (*p* = 0.67). The ablation method (cryo vs. RF) did not significantly affect QoL, F(1, 80) = 1.33, *p* = 0.47, indicating no significant difference in QoL outcomes between patients undergoing cryo and RF. The interaction between the ablation method and time was insignificant, F(2, 150) = 0.85, *p* = 0.45, suggesting that the improvement in QoL over time was similar in both treatment groups. None of the covariates predicted QoL improvement, including age (B = −0.03, SE = 0.02, *p* = 0.42), rhythm control (B = 0.30, SE = 0.26, *p* = 0.18), hypertension (B = 0.29, SE = 0.91, *p* = 0.19), Indexed left atrial volume (mL/m^2^) (B = −0.2, SE = 0.29, *p* = 0.36), (B = −1.50, SE = 2.05, *p* = 0.46), BMI (B = 0.07, SE = 0.09, *p* = 0.46), IHD (B = −0.61, SE = 1.57, *p* = 0.72), CCF (B = −0.72, SE = 1.10, *p* = 0.32), or additional ablations (B = 0.95, SE = 1.31, *p* = 0.48).

The random intercepts model showed substantial individual variability (*p* < 0.01), confirming that baseline QoL varied between patients. However, the random effects did not alter the main finding that QoL improved over time regardless of treatment type.

## 4. Discussion

This is the first study to assess and compare QoL between RF and cryo with and without additional ablations for 30 months following PVI for PAF. The study proposes three main findings. First, QoL after RF and cryo improved significantly at 12 months, without further improvement up to 30 months following the procedure. Second, RF and cryo caused similar QoL improvement in the PAF cohort. Third, additional ablation did not cause further QoL improvement compared to patients with only PVI. It is important to note that additional ablations were conducted in the same setting and used the same ablation mode as the index procedure.

The primary goal in managing AF is to improve symptoms, decrease disability, lower healthcare resource utilisation (emergency department visits and hospitalisations), and enhance QoL [19]. While traditional measures like arrhythmia-free survival are essential for comparing treatments, they may not fully capture the overall effects of different approaches. For example, even if AF recurs, the absence of symptoms or a reduction in symptomatic AF episodes can be considered a successful outcome [20]. The use of generic instruments does have its advantages, as they are extensively validated across various populations and disease conditions. However, these instruments tend to focus more on general physical health and functionality, which may make them less effective in capturing AF-specific QoL.

Conversely, disease-specific instruments may not allow for comparison between different disease states. Still, they are more precise in measuring QoL domains directly relevant to AF, making them more responsive to patient health status changes.

Table 2 summarises studies that examined the improvement in QoL after PVI compared to other rhythm control modalities. RF and cryo are suggested to improve QoL in the short and long term [6,21,22,23,24,25,26,27,28,29,30,31,32,33,34,35,36,37], which agrees with this study’s findings. Furthermore, PVI was superior to AADs in QoL in these patients [38,39,40,41,42,43]. The improvement is related to the drop in AF burden, which was suggested by the CIRCA-DOSE trial, which used continuous rhythm monitoring to assess the burden after RF and cryo [44].

This study found improved mental and physical quality of life 12 months following the index ablation, sustained throughout the 30-month follow-up period. This was observed in both ablation methods, and there was no statistically significant difference between the two groups. The substantial improvement at 12 months aligns with reducing AF symptoms and clinical procedure success in our study. This is also in keeping with the Fire and Ice QoL analysis, which suggested that the QoL improvement was sustained from 6 to 30 months post-PVI [8]. However, it is important to note that the AFEQT, EQ-VAS, and EQ-5D-3L surveys may not be precise enough when evaluating patient performance based on ablation methods, mainly due to the relatively short follow-up periods (less than 5–10 years) when comparing the groups [45]. In addition, the EQ-5D-3L, which uses a three-level scoring system for easy scoring, lacks specificity compared to more detailed questionnaires and may be subject to a ceiling effect [46,47]. Therefore, future studies should assess QoL over a more extended period.

**Table 2:** Studies that examined QoL improvement after PVI alone and in relation to other rhythm control modalities.

Author and Year	n	AF	Questionnaire	Follow-Up (months)	Treatment	QoL Improvement
PVI only						
(Pappone et al., 2003) [6]	1171	PAF (70%)	SF-36	12	RF	Yes
(Goldberg et al., 2003) [21]	33	PAF	SF-36	36	RF	Yes
(Erdogan et al., 2003) [22]	30	PAF	SF-36, SSC	36	RF	Yes
(Pürerfellner et al., 2004) [23]	89	PAF	SF-36	6	RF	Yes
(Weerasooriya et al., 2005) [24]	63	PAF	SF-36	12	RF	Yes
(Fiala et al., 2014) [25]	160	PersAF	EQ-5D	12	RF	Yes
(Mohanty et al., 2014) [26]	62	Asymptomatic Permanent AF	SF-36	20	RF	Yes (if successful)
(Mantovan et al., 2013) [27]	100	PAF (64%)	SF-36	12	PVI	No
(Wokhlu et al., 2010) [28]	502	PAF (51%)	SF-36	48	RF	Yes
(Boveda et al., 2018) [29]	130	PersAF	SF-36	12	cryo	Yes
(Natale et al., 2021) [30]	333	PersAF	AFEQT	15	RF	Yes
(Fichtner et al., 2012) [31]	133	PAF	WHO5, MDI, SV, VE	52	PVI	Yes
(Boersma et al., 2020) [32]	1054	PAF (70%)	AFEQT	12	RF	Yes
(Terricabras et al., 2020) [33]	549	PersAF	EQ-5D-3L	18	PVI	Yes
(Su et al., 2020) [34]	165	PersAF	AFEQT, SF-12	12	Cryo	Yes
(Jain et al., 2020) [36]	319	PAF	SF-36	36	Cryo	Yes
(Onishi et al., 2020) [35]	45	Asymptomatic PersAF	AFQLQ	12	PVI	Yes
(Gupta et al., 2021) [37]	329	PAF	AFEQT, EQ-5D-5L	12	RF	Yes
PVI vs. AAD						
(Pavlovic et al., 2020) [38]	218 (RF = 109)	PAF	AFEQT, SF-36	12	Cryo vs. AAD	Cryo > AAD
(Jaïs et al., 2008) [39]	112 (RF = 53)	PAF	SF-36	12	RF vs. AAD	RF > AAD
(Reynolds et al., 2010) [40]	159 (RF = 103)	PAF	SF-36	12	RF vs. AAD	RF > AAD
(Walfridsson et al., 2015) [41]	294 (RF = 146)	PAF	SF-36, EQ	24	RF vs. AAD	RF > AAD
(Blomström-Lundqvist et al., 2019) [42]	155 (PVI = 75)	PAF (73%)	SF-36	12	PVI vs. AAD	PVI > AAD
(Mark et al., 2019) [43]	2204 (PVI = 1108)	PAF (57%)	AFEQT, MAFSI	60	PVI vs. AAD	PVI > AAD
Radiofrequency ablation vs. cryoballoon ablation						
(Andrade et al., 2020b) [48]	346 (RF = 173)	PAF	HRQOL	12	RF vs. cryo	RF = cryo
(Kuck et al., 2016c) [8]	750 (RF = 376)	PAF	SF-12, EQ-5D	30	RF vs. cryo	RF = cryo
(Malmborg et al., 2013) [49]	110 (RF = 56)	PAF (31%)	SF-36	12	RF vs. cryo	RF = cryo
PVI vs. surgical ablation						
(Haldar et al., 2020) [50]	120 (PVI = 60)	Permanent AF	EQ-6D-5L, AFEQT, HEQ	12	PVI vs. Surgical ablation	PVI > surgical ablation

AFEQT (the AF effect on quality of life survey). RF: radiofrequency ablation. Cryo: cryoballoon ablation. PAF: paroxysmal atrial fibrillation. PersAF: persistent atrial fibrillation. PVI: paroxysmal atrial fibrillation. AAD: antiarrhythmic drug.

Our results suggested that ablations outside PV did not provide additional QoL benefits up to 30 months following ablation compared to patients with PVI alone in RF and cryo. The higher rate of additional ablations outside of the PVs in RF reflects the procedural differences between the two ablation modalities. RF, being point-by-point, may result in non-durable or incomplete lesion sets, leading to more frequent additional ablations outside of PVs. On the other hand, cryo provides a more consistent circumferential PVI, likely decreasing the need for further ablations. While additional ablations outside PVs may offer incremental benefits in reducing AF recurrence in selected patients [51], this does not consistently translate into improved QoL compared to PVI alone. This occurred similarly in RF and cryo, although RF patients had significantly more ablations outside the PVs than cryo patients who only had roofline ablations using the cryo catheter. The reasons are multifactorial, encompassing the complexity of AF mechanisms, patient-specific factors, procedural risks, and the intricate relationship between symptom burden, AF recurrence, and patient-perceived QoL. These findings underscore the importance of individualised treatment strategies, focusing on patient-centred outcomes and carefully selecting patients for extensive ablation procedures. Further research is warranted to refine patient selection criteria and develop more precise QoL measurement tools tailored to AF populations.

## 5. Conclusions

In patients undergoing their first ablation, PAF, RF, and cryo caused similar QoL improvement at 12 months. The improvement was sustained up to 30 months after a single procedure. Additional ablations did not provide additional QoL improvement to sole PVI.

## 6. Limitations

This is a single-centre retrospective study with AF recurrence detected using 12-lead ECG or Holter monitoring. Long-term monitoring was not performed, and the AF burden was not evaluated in all patients. Thus, sub-clinical and micro-AF episodes could have been missed. Furthermore, the AF burden may not be correlated with QoL with or without additional ablations. We cannot rule out the possibility of “expectation bias” or the placebo effect contributing to the significant improvement in QoL observed from the baseline assessment. Although the study sample is relatively small, a post hoc analysis yielded a power of 84% to detect a significant difference in QoL.

Furthermore, patients stopping their antiarrhythmic drugs were included in the analysis. Future studies must match patients with antiarrhythmic drugs and their cessation to limit confounding. AF burden after PVI was not calculated through long-term monitoring, which limited correlation with QoL. There were significantly fewer additional ablations in cryo than in RF. This imbalance limits the ability to draw meaningful conclusions about the impact of additional ablations on QoL outcomes in cryo.

Consequently, the findings regarding the effect of additional procedures predominantly apply to the RF group, which reduces the novelty and generalizability of the results for the cryo cohort. Future studies with a larger cohort of cryoballoon patients undergoing repeat ablations are necessary to assess QoL outcomes in this population fully. Furthermore, in future studies, it will be essential to conduct a multi-centre, randomised, controlled trial with a larger sample size to enhance the generalizability of the findings. Equalising group sizes between cryo and RF will allow for more robust comparisons and reduce potential biases. Standardising ablation techniques and lesion placements across treatment groups will also help mitigate heterogeneity and facilitate clearer interpretations of the outcomes. For example, CFAE was utilised in our PAF cohort, although the evidence states it does not add further clinical benefits to PVI [52].

## Figures and Tables

**Table 1:** The study’s demographics and ablation details.

	Total	RF	Cryo	*p*-Value
Age at procedure	62.1 ± 4.2	59.6 ± 1.8	64.5 ± 2.7	0.74
Male	144 (70%)	85 (67%)	59 (74%)	0.65
Diabetes melitus	13 (6%)	9 (7%)	4 (5%)	0.47
Cerebrovascular event	6 (3%)	4 (3%)	3 (3%)	0.85
Chronic cardiac failure	16 (11%)	12 (13%)	4 (8%)	0.32
Ischemic heart disease	19 (9%)	10 (8%)	9 (11%)	0.77
Hypertension	75 (36%)	44 (35%)	31 (39%)	0.63
Indexed left atrial volume (mL/m^2^)	32.3 ± 4	31 ± 2.4	33.2 ± 2.5	0.41
Body mass index (kg/m^2^)	26.3 ± 2.5	28.5 ± 4.1	24.2 ± 3.2	0.08
Ablations outside pulmonary veins	74 (36%)	52 (41%)	22 (28%)	0.01
Medications details				
Total AADs	133 (64%)	86 (68%)	47 (59%)	0.09
Flecainide	29 (14%)	19 (15%)	10 (13%)	0.74
Sotalol	75 (36%)	49 (39%)	26 (33%)	0.4
Amiodarone, dronedarone	29 (14%)	18 (14%)	11 (14%)	0.91
Time AAD stopped after PVI	6.4 ± 2.3	6 ± 2.2	6.8 ± 2.3	0.28
Long-term AADs	101 (49%)	60 (47%)	41 (51%)	0.62
Total rate control	121 (58%)	78 (61%)	43 (54%)	0.32
Betablockers	101 (49%)	61 (48%)	40 (50%)	0.8
Diltiazem, verapamil	14 (7%)	9 (7%)	5 (7%)	0.89
Rate control on long term	120 (78%)	78 (61%)	42 (53%)	0.11
Ablation outcomes				
Success at one year	157 (76%)	98 (77%)	59 (74%)	0.63
Success at 30 months	143 (69%)	87 (69%)	56 (70%)	0.93

PVI: pulmonary vein isolation. AAD: antiarrhythmic drug.

## Data Availability

The raw data supporting the conclusions of this article will be made available by the authors on request.

## Supplementary Materials

The following supporting information can be downloaded at: https://www.mdpi.com/article/10.3390/jcdd11120385/s1, Figure S1: Details of the utilized questionnaires questionnaires.
