# Supplementary material for: Long-Term Patient-Reported Outcomes After Radiofrequency Ablation and Cryoballoon Ablation for Paroxysmal Atrial Fibrillation: The Effect of Additional Ablations
Source: J Cardiovasc Dev Dis. 2024 Nov 30;11(12):385. doi: 10.3390/jcdd11120385 (PMC11676108; doi:10.3390/jcdd11120385)

Figure S1: Details of the utilised questionnaires

You are having an ablation or have already had your ablation as part of your treatment for AF and we would like to assess the difference this will make to your symptoms. We are asking you to complete these questionnaires to help us do this and assist with the options for your future treatments.

We will see you in the follow-up clinic at 3, 6, and (12) months after your procedure and will be asking you to complete the same questionnaires this wil enable us to be able to monitor your recovery and progress.

Thank-you for taking the time to complete these questionnaires, once you have finished them please hand them in to the Specialist Nurse or Clinic Co-ordinator.

Many thanks

Dr Peter Stafford	Dr Andre Ng	Dr Alastair Sandilands	Dr Riyaz Somani
Consultant Electrophysiologist	Consultant Electrophysiologist	Consultant Electrophysiologist	Consultant Electrophysiologist

Sue Armstrong
Cardiac Rhythm Nurse Specialist

Office use only:

Date of preadm — Co-morbities IHD, Resp disease Thyroid

TCI date — CVA / TIAs Diabetes Hypertension

Age at ablation — other please list:

Procedure / score / mapping system

Consultant — EHRA score — NYHA score

BMI

Atrial Fibrillation Effect on QualiTy-of-life (AFEQT) Questionnaire

Section 1. Occurrence of atrial fibrillation **Name or ID:**______________

Are you currently in atrial fibrillation? ☐ Yes ☐ No

If No, when was the last time you were aware of having had an episode of atrial fibrillation? (Please check one answer which best describes your situation)

_earlier today — _1 month to 1 year ago
_within the past week — _ more than 1 year ago
_within the past month — _I was never aware of having atrial fibrillation

Section 2. The following questions refer to how atrial fibrillation affects your quality of life.
On a scale of 1 to 7, over the past 4 weeks, as a result of your atrial fibrillation, how much were you bothered by:
(Please circle one number which best describes your situation)

	Not at all bothered Or I did not have this symptom	Hardly bothered	A little bothered	Moderately bothered	Quite a bit bothered	Very bothered	Extremely bothered
1. Palpitations: Heart fluttering, skipping or racing	1	2	3	4	5	6	7
2. Irregular heart beat	1	2	3	4	5	6	7
3. A pause in heart activity	1	2	3	4	5	6	7
4. Lightheadedness or dizziness	1	2	3	4	5	6	7

On a scale of 1 to 7, over the past 4 weeks, have you been limited by your atrial fibrillation in your:
(Please circle one number which best describes your situation)

	Not at all limited	Hardly limited	A little limited	Moderately limited	Quite a bit limited	Very limited	Extremely limited
5. Ability to have recreational pastimes, sports, and hobbies	1	2	3	4	5	6	7
6. Ability to have a relationship and do things with friends and family	1	2	3	4	5	6	7

On a scale of 1 to 7, over the past 4 weeks, as a result of your atrial fibrillation, how much difficulty have you had in:
(Please circle one number which best describes your situation)

	No difficulty at all	Hardly any difficulty	A little difficulty	Moderate difficulty	Quite a bit of difficulty	A lot of difficulty	Extreme difficulty
7. Doing any activity because you felt tired, fatigued, or low on energy	1	2	3	4	5	6	7
8. Doing physical activity because of shortness of breath	1	2	3	4	5	6	7
9. Exercising	1	2	3	4	5	6	7
10. Walking briskly	1	2	3	4	5	6	7
11. Walking briskly uphill or carrying groceries or other items, up a flight of stairs without stopping	1	2	3	4	5	6	7
12. Doing vigorous activities such as lifting or moving heavy furniture, running, or participating in strenuous sports like tennis or racquetball	1	2	3	4	5	6	7

Version 1.0 © 2009 St. Jude Medical, Inc. All Rights Reserved License Required for Use 1

On a scale of 1 to 7, over the past 4 weeks as a result of your atrial fibrillation, how much did the feelings below bother you? (Please circle one number which best describes your situation)

	Not at all Bothered	Hardly bothered	A little bothered	Moderately bothered	Quite a bit bothered	Very bothered	Extremely bothered
13. Feeling worried or anxious that your atrial fibrillation can start anytime	1	2	3	4	5	6	7
14. Feeling worried that atrial fibrillation may worsen other medical conditions in the long run	1	2	3	4	5	6	7

On a scale of 1 to 7, over the past 4 weeks, as a result of your atrial fibrillation treatment, how much were you bothered by: (Please circle one number which best describes your situation)

	Not at all bothered	Hardly bothered	A little bothered	Moderately bothered	Quite a bit bothered	Very bothered	Extremely bothered
15. Worrying about the treatment side effects from medications	1	2	3	4	5	6	7
16. Worrying about complications or side effects from procedures like catheter ablation, surgery, or pacemakers therapy	1	2	3	4	5	6	7
17. Worrying about side effects of blood thinners such as nosebleeds, bleeding gums when brushing teeth, heavy bleeding from cuts, or bruising.	1	2	3	4	5	6	7
18. Worrying or feeling anxious that your treatment interferes with your daily activities	1	2	3	4	5	6	7

On a scale of 1 to 7, overall, how satisfied are you **at the present time** with:
(Please circle one number which best describes your situation)

	Extremely satisfied	Very satisfied	Somewhat satisfied	Mixed with satisfied and dissatisfied	Somewhat dissatisfied	Very dissatisfied	Extremely dissatisfied
19. How well your current treatment controls your atrial fibrillation?	1	2	3	4	5	6	7
20. The extent to which treatment has relieved your symptoms of atrial fibrillation?	1	2	3	4	5	6	7

By placing a tick in one box in each group below, please indicate which statements best describe your own health state today.

Mobility
I have no problems in walking about ☐
I have some problems in walking about ☐
I am confined to bed ☐

Self-Care
I have no problems with self-care ☐
I have some problems washing or dressing myself ☐
I am unable to wash or dress myself ☐

Usual Activities *(e.g. work, study, housework, family or leisure activities)*
I have no problems with performing my usual activities ☐
I have some problems with performing my usual activities ☐
I am unable to perform my usual activities ☐

Pain/Discomfort
I have no pain or discomfort ☐
I have moderate pain or discomfort ☐
I have extreme pain or discomfort ☐

Anxiety/Depression
I am not anxious or depressed ☐
I am moderately anxious or depressed ☐
I am extremely anxious or depressed ☐

To help people say how good or bad a health state is, we have drawn a scale (rather like a thermometer) on which the best state you can imagine is marked 100 and the worst state you can imagine is marked 0.

We would like you to indicate on this scale how good or bad your own health is today, in your opinion. Please do this by drawing a line from the box below to whichever point on the scale indicates how good or bad your health state is today.

Your own health state today

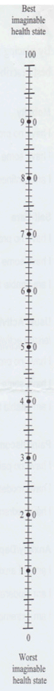

Supplement: Supplementary file 1 [file jcdd-11-00385-s001.zip › jcdd-3224147-supplementary.pdf]
